# A comprehensive analysis of renal cell carcinoma as first and second primary cancers

**DOI:** 10.1186/s12957-022-02493-6

**Published:** 2022-02-27

**Authors:** Jinchao Chen, Jianmin Lou, Yedie He, Zhenjie Zhu, Shaoxing Zhu

**Affiliations:** Department of Urologic Surgery, The Cancer Hospital of the University of Chinese Academy of Sciences, Zhejiang Cancer Hospital, Institute of Basic Medicine and Cancer (IBMC), Chinese Academy of Sciences, No 1, East Banshan Road, Gongshu District, Hangzhou, 310022 People’s Republic of China

**Keywords:** Renal cell carcinoma, Multiple primary malignancy, Second primary cancers, Comparison

## Abstract

**Objective:**

Second primary renal cell carcinoma (2nd RCC) refers to renal cell carcinoma (RCC) diagnosed after another unrelated malignancy. This study aims to compare the clinical manifestation, pathology, treatment, and prognostic features of patients with 2nd RCC and first primary renal cell carcinoma (1st RCC).

**Materials and methods:**

Data of the patients with localized RCC were retrospectively collected. They were classified as 2nd RCC or 1st RCC according to a previously diagnosed cancer, including 113 cases of 2nd RCC and 749 cases of 1st RCC.

**Results:**

The most common types of extrarenal malignancies in patients with 2nd RCC include lung, colorectal, breast, gynecological, and gastric cancers. The age and smoking rate of 2nd RCC patients were significantly higher than in those of 1st RCC patients. For 2nd RCC patients, fewer had clinical symptoms and renal masses tend to be smaller. One hundred and eight (95.6%) patients with 2nd RCC received surgical interventions. All patients with 1st RCC underwent renal surgery. More patients with 2nd RCC underwent a partial nephrectomy. Pathologically, there was no significant difference in postoperative pathological types between the 2nd and 1st RCCs. However, the 2nd RCCs were commonly identified in the early stages. The median overall survival (OS) of 2nd RCC patients was 117 months, which was shorter than that of 1st RCC patients.

**Conclusions:**

Second RCC is not uncommon. More attention should be paid to screening for 2nd RCC in cancer survivors. There are some differences between patients with 2nd and 1st RCCs that should be viewed separately.

**Supplementary Information:**

The online version contains supplementary material available at 10.1186/s12957-022-02493-6.

## Introduction

A second primary malignancy (SPM) refers to a malignant tumor found at the same time as or after a primary malignancy, excluding metastasis and recurrence [1]. The advances in cancer diagnosis and treatment have not only prolonged the life of cancer survivors but also increased the diagnosis of SPM [2]. SPM is estimated to be the sixth most common malignancy in the world [3, 4]. Its occurrence reflects potential immune deficiency, gene mutation, carcinogen exposure (drinking, smoking), or the sequelae of cancer and its treatment (radiotherapy and chemotherapy) [5]. Therefore, improving the diagnosis and treatment of SPM is important for the long-term management of cancer. We also need to pay attention to identify the environmental, behavioral, and genetic factors affecting SPM. In so doing, the risk of SPM may be reduced by controlling the related factors [6].

Renal cell carcinoma (RCC) is one of the most common malignancies of the urinary system, with an increasing incidence in recent years, but the overall mortality rate has stabilized [7]. Non-invasive imaging examinations, such as B-ultrasound and computer tomography (CT), have been widely used to increase the detection of renal cancers in the early stages [8]. RCC may be a first primary cancer (first primary RCC, 1st RCC), or it can occur as a second primary RCC (2nd RCC) in patients with a previous history of malignancy. 2nd RCC is one of the most common SPM, with an incidence of 10.9–28.9% [1, 9, 10].

Other types of primary malignancies associated with RCC include bladder, prostate, colorectal, lung, cutaneous malignant melanoma (MM), and non-Hodgkin’s lymphoma (NHL) [10]. Although it is not uncommon, there are few studies on the clinical features, diagnosis, treatment, and prognosis of the 2nd RCC. Furthermore, there is also a lack of comparative studies between 2nd RCC and 1st RCC.

By collecting the data of patients with 2nd RCC, we systematically analyzed the clinical features, diagnosis, treatment, and prognosis and compared them with those of the 1st RCC patients treated in the same period.

## Materials and methods

Studies involving human participants were reviewed and approved by the Medical Ethics Committee of Zhejiang Cancer Hospital. The database records of patients diagnosed with RCC at Zhejiang Cancer Hospital from January 2009 to June 2019 were reviewed. Patients with distant metastases were excluded. The patients in the cohort were classified as having 2nd or 1st RCC according to their cancer history. Non-renal primary malignancies were recorded and classified as antecedent and synchronous, and the patients who were diagnosed after RCC were excluded. Synchronous malignancies were defined as those diagnosed concurrently or within 6 months of the initial diagnosis.

We retrospectively collected 113 cases of 2nd RCC and 749 cases of 1st RCC. The clinical data of these patients were analyzed, including demographic data, pathology and treatment of non-renal malignancies, symptoms, imaging features, pathology, and treatment of the RCC (2nd RCC and 1st RCC). The symptoms included flank pain, visible hematuria, palpable abdominal, paraneoplastic syndromes, and symptoms caused by metastatic disease, such as bone pain or persistent cough. The puncture or surgical specimens were evaluated by specialized pathologists. Patient follow-up ensured collection of data pertaining to recurrence rate, metastasis, and survival status. The overall survival (OS) was defined as the time from the diagnosis of RCC to the last follow-up or death. Progression-free survival (PFS) was defined as the time from diagnosis to the first documentation of progressive disease or death, whichever occurred first. Cancer-specific survival (CSS) was defined as the time from the date of diagnosis to the date of death caused by RCC. The pathological stage and histological grade were defined according to the International Union Against Cancer TNM classification [11].

Categorical variables and continuous variables were tested using the *χ*^2^ test and *t*-test, respectively. OS, PFS, and CSS were calculated using the Kaplan-Meier method for the study population. Multivariate Cox regression analysis was used to explore factors influencing prognosis. All statistical analyses were performed using IBM SPSS Statistics for Windows, version 19 (IBM Corp., Armonk, NY, USA). Statistical significance was set at *p* < 0.05.

## Results

### Demographics and clinical presentations of 2nd RCC and 1st RCC

The demographic details of the 2nd RCC and 1st RCC groups are shown in Table 1. Patients with 2nd RCC were older in age at diagnosis (58.2 vs. 55.0 years, *p* = 0.008) and had a higher rate of smoking (54.1% vs. 35.0%, *p* = 0.036). The rate of family cancer history was slightly higher in patients with 2nd RCC, but the difference was not significant (31.9% vs. 23.9%, *p* = 0.068). There was no significant difference in sex distribution or body mass index (BMI) between the two groups. Of the patients with 1st RCC, 29.2% had symptoms such as lower back pain and hematuria. The vast majority (94.9%) of the 2nd RCC were detected incidentally with no symptoms and there was a significant difference between the two groups (*p* < 0.001).

**Table 1:** Demographics and clinical characteristics of patients with second primary renal cell carcinoma (2nd RCC) and first primary renal cell carcinoma (1st RCC)

Characteristic		2nd RCC	1st RCC	*p* value
Patients				
Number of patients		113	749	
Gender	Male	67 (59.3%)	452 (60.3%)	0.83
	Female	46 (40.7%)	297 (39.7%)	
Mean age, years (±*SD*)		58.2 (±10.2)	55.0 (±12.1)	0.008
Smoking history	Yes	51 (45.1%)	262 (35.0%)	0.036
	No	62 (54.9%)	487 (65.0%)	
Family history of tumor	Yes	36 (31.9%)	179 (23.9%)	0.068
	No	77 (68.1%)	570 (76.1%)	
Mean BMI, kg/m^2^ (±*SD*)		23.9 (±3.2)	23.9 (±3.4)	1.00
Disease				
Symptom	Yes	2 (1.8%)	219 (29.2%)	<0.001
	No	111 (98.2%)	530 (70.8%)	
Synchronous		71 (62.8%)	–	–
Metachronous		42 (37.2%)	–	
Mean interval between first extrarenal malignancies and renal cell carcinoma, months (range)		33.9 (0–540)	–	–
Mean diameter of renal mass, cm (±*SD* )		3.6 (±1.6)	4.7 (±2.5)	<0.001
Renal surgery	Radical nephrectomy	43 (39.8%)	394 (52.6%)	0.013
	Partial nephrectomy	65 (60.2%)	355 (47.4%)	

*RCC* renal cell carcinoma, *BMI* body mass index

### Origin, staging, and primary treatment of non-renal malignancies

The most common primary non-renal malignancies of 2nd RCC were lung cancer (20.4%), followed by colorectal (14.2%), breast (10.6%), gynecological (9.7%), thyroid (8.8%), gastric (8.0%), nasopharyngeal (8.0%), and esophageal (4.4%) cancers. Patients with stage I–II non-renal malignant tumors accounted for 76.7%, and 45.1% of the patients had a history of radiotherapy or chemotherapy (Supplemental Table 1). Synchronous 2nd RCC was found in 71 patients (62.8%), and antecedent 2nd RCC accounted for 37.2%, with a mean interval between the two cancer sets of 33.9 months.

### Diagnosis, treatment, and pathology of 2nd RCC and 1st RCC

Comparing patients with 2nd RCC to patients with 1st RCC, the former had significantly smaller tumor size (3.6 cm vs. 4.7 cm, *p* < 0.001). Five patients (4.4%) with suspected 2nd RCC underwent renal puncture biopsy, and all the pathological results indicated RCC. None of the patients with 1st RCC had a renal biopsy.

Of the 113 patients with 2nd RCC, 108 (95.6%) nephrectomies were performed including 65 partial (60.2%) and 43 radical nephrectomies (39.8%), 4 (3.5%) patients received observation, and 1 (0.9%) patient underwent ablation. The reasons for non-surgical treatment for these patients included advanced stage (4 cases) and postoperative complications (1 case) of the non-renal malignancies. All patients with 1st RCC underwent nephrectomy, including partial nephrectomy (*n* = 355, 47.4%) and radical nephrectomy (*n* = 394, 52.6%). There was a significant difference in the treatment mode between the two groups (Table 1).

When comparing synchronous 2nd RCC with metachronous 2nd RCC, there was no significant difference in age, sex ratio, smoking history, family history of tumor, BMI, and diameter of renal mass. However, in terms of the type of surgery, more patients with metachronous 2nd RCC received partial nephrectomy than patients with synchronous 2nd RCC (*p* = 0.01).

The postoperative pathological categories of patients with 2nd and 1st RCCs are shown in Table 2. There was no significant difference in the distribution of pathological types between the two groups (*p* = 0.982). In terms of pathological stages of the RCC, more patients with 2nd RCC had T1 RCC compared with patients with 1st RCC (91.5% vs. 79.4%, *p* < 0.001). However, there was no significant difference between the two groups in terms of nuclear grade and single/multiple lesions.

**Table 2:** Pathological characteristics of patients with second primary renal cell carcinoma (2nd RCC) and first primary renal cell carcinoma (1st RCC)

		2nd RCC	1st RCC	*p* value
Pathological type	Clear cell RCC	98 (86.7%)	630 (84.1%)	0.98
	Papillary RCC	6 (5.3%)	44 (5.9%)	
	Chromophobe RCC	5 (4.4%)	51 (6.8%)	
	Translocation RCC	1 (0.88%)	4 (0.53%)	
	Sarcomatoid variants of RCC	1 (0.88%)	3 (0.4%)	
	Renal medullary carcinoma	0	1 (0.13%)	
	Renal collecting duct carcinoma	0	1 (0.13%)	
	Mucinous tubular and spindle cell carcinoma	0	1 (0.13%)	
	Unclassified RCC	2 (1.8%)	14 (1.9%)	
Single/multiple		113/0	744/5	0.38
Pathological grade	I	16 (22.9%)	104 (24.9%)	0.45
	II	44 (62.9%)	236 (56.6%)	
	III	10 (14.3%)	69 (16.5%)	
	IV	0	8 (1.9%)	
Pathological stage	T1	97 (91.5%)	595 (79.4%)	<0.001
	T2	2 (1.9%)	86 (11.5%)	
	T3	7 (6.6%)	50 (6.7%)	
	T4	0	7 (0.9%)	
	TxN1	0	11 (1.5%)	

*RCC* renal cell carcinoma

### Outcomes of 2nd RCC and 1st RCC

**Fig. 1:**
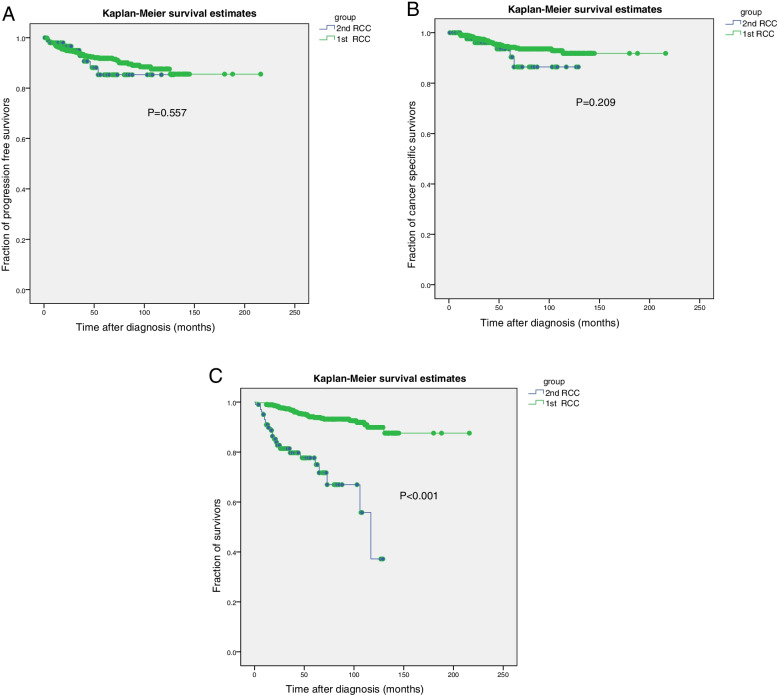
Kaplan-Meier curves for progression-free survival (PFS) (**A**), cancer-specific survival (CSS) (**B**), and overall survival (OS) (**C**) in patients with second primary renal cell carcinoma (2nd RCC) and first primary renal cell carcinoma (1st RCC)

One hundred and two patients with 2nd RCC and 720 patients with 1st RCC were followed up for a median period of 52 months (range,1–216 months). For patients with 2nd RCC, metastasis occurred in 8 patients with a median progression time of 32 months (4–54 months), and the diagnosis was confirmed by puncture biopsy in six patients and by combining imaging and clinical features in the other two patients. At the last follow-up, 78 (76.5%) patients were still living and 24 (23.5%) patients had died. Among those who had died, non-renal malignancies were the cause in 18 patients (75%). The median OS was 117 months (95% confidence interval [*CI*] = 97.16–136.84) for patients with 2nd RCC. For patients with 1st RCC, metastasis occurred in 61 patients, with a median progression time of 18 months (range, 3–126 months). At the last follow-up, 680 patients (94.4%) had survived and 40 patients (5.6%) had died. Of those who had died, 35 patients died of RCC and 5 patients died of other causes. The OS of the 1st RCC was significantly longer than that of the 2nd RCC (*p* < 0.001). However, there was no significant difference in PFS and CSS between the two groups (*p* = 0.0.557 and *p* = 0.209, respectively) (Fig. 1).

Univariate analysis showed that sex, symptoms, stage of RCC, and renal surgery were independent prognostic factors of OS in patients with 2nd RCC (Table 3). In contrast, age, symptoms, and the stage of RCC were independent prognostic factors of OS in patients with 1st RCC (Table 4). Results of the multivariable analysis indicated that OS was improved among female patients with 2nd RCC (Table 3). Young patients with no symptoms and an early stage of RCC had better OS in patients with 1st RCC (Table 4).

**Table 3:** Univariate survival analysis and multivariable analysis of patients with second primary renal cell carcinoma (2nd RCC)

Overall survival	Univariate analysis			Multivariable analysis		
	*HR*	95% *CI*	*p* value	*HR*	95% *CI*	*p* value
Age (≥ 60)	1.044	0.488–2.234	0.912	0.599	0.246–1.459	0.259
Gender (male)	3.897	1.462–10.388	0.007	5.065	1.400–18.332	0.013
Smoking history (yes)	1.653	0.763–3.580	0.202	0.721	0.244–2.133	0.555
Family history of tumor (yes)	0.972	0.420–2.245	0.946	0.531	0.201–1.405	0.202
Primary non-renal malignancy (lung cancer)	0.961	0.385–2.394	0.931	1.000	0.349–2.859	0.999
Symptoms (yes)	7.073	2.354–21.251	<0.001	5.217	0.726–37.501	0.101
Interval between first extrarenal malignancies and renal cell carcinoma (metachronous)	0.685	0.297–1.582	0.376	0.719	0.278–1.862	0.497
Renal surgery (yes)	0.151	0.05–0.457	0.001	0.329	0.069–1.578	0.165
Stage of RCC (≥T2)	7.577	2.156–26.627	0.002	1.211	0.154–9.493	0.856

*RCC* renal cell carcinoma

**Table 4:** Univariate survival analysis and multivariable analysis of patients with first primary renal cell carcinoma (1st RCC)

Overall survival	Univariate analysis			Multivariable analysis		
	*HR*	95% *CI*	*p* value	*HR*	95% *CI*	*p* value
Age (≥ge years)	2.923	1.550–5.511	0.001	2.839	1.486–5.422	0.002
Gender (male)	1.100	0.587–2.059	0.766	1.684	0.691–4.101	0.252
Smoking history (yes)	1.451	0.775–2.716	0.245	2.034	0.836–4.948	0.118
Family history of tumor (yes)	0.869	0.400–1.889	0.723	.870	0.398–1.903	0.728
Symptoms (yes)	2.667	1.432–4.967	0.002	1.894	0.988–3.630	0.054
Stage of RCC (≥T2)	5.742	3.064–10.759	<0.001	4.561	2.368–8.783	<0.001

## Discussion

RCC has been frequently described as a second cancer event following the diagnosis of other primary cancers [12]. Although there have been reports relating to the increased risk of 2nd RCC in cancer survivors, there have been few studies that investigated how 2nd RCC was different from 1st RCC. Thus, our study compared the clinical features, diagnosis, treatment, and prognosis of 2nd and 1st RCCs. To our knowledge, this study is the largest and most comprehensive profile of this clinical scenario.

Sato et al. reported that RCC patients with other primary malignancies were approximately 5 years older than those without [9]. Other SPM studies have also shown similar results. Jo et al. reported that the age at diagnosis of 2nd pancreatic cancers was older than that of 1st pancreatic cancers [2]. Our study shared similar results. It is well known that the incidence of epithelial cancers generally increased with age [13]. The age at diagnosis may differ because patients with first primary cancers have lived long enough to develop another primary cancer.

Previous studies have confirmed that smoking is a high-risk factor for RCC and multiple malignancies [8, 14]. In our study, it was found that the smoking rate of patients with 2nd RCC was higher than that of patients with 1st RCC. Previous studies have also found that continued smoking is considered the strongest adverse predictor among cancer survivors [15]. Conversely, smoking cessation after the first cancer diagnosis prolongs the time before a new malignancy develops, as well as the total survival time [15, 16]. Therefore, it provides the evidence for clinicians in supporting patients to stop smoking, especially for cancer survivors.

Obesity is also a risk factor for many malignancies [17, 18]. Unlike smoking, this study found that there was no significant difference in the BMI of patients with 2nd RCC versus 1st RCC. This finding suggests that smoking is a higher risk factor for the development of 2nd RCC than obesity. More studies are needed to explore whether weight loss can reduce the risk of SPM.

Abdel-Rahman et al. found that the diagnosis of 2nd RCC usually occurs within 5 years after the first primary malignancy [12]. In our study, 81.4% of 2nd RCC cases occurred within 5 years of the primary tumor diagnosis, which is consistent with previous studies. It is worth noting that synchronous 2nd RCC accounted for 61.9% in our study. The rate is higher than the 34.6% reported by Beisland but similar to the 59.4% reported by Sato [9, 10]. This result suggests that we need to carry out a systematic examination before the treatment of patients with RCC to prevent the risk of missing malignant tumors in other parts of the body. However, our study also found that 18.6% of 2nd RCC cases occurred 5 years after the first primary malignancies, which revealed that there may be two peaks in the incidence of 2nd RCC, namely, within 6 months and 5 years after the initial diagnosis. This is similar to the timeframe reported by Czene and Hemminki [19]. Long-term follow-up may therefore be needed after the treatment of malignancies to detect not only the recurrence and metastasis of primary malignancies but also the 2nd RCC.

With greater access to imaging examinations, more than 60% of patients are diagnosed incidentally [11]. In our study, it was found that patients with 2nd RCC presented fewer symptoms, and the tumor size and postoperative pathological stage in patients with 2nd RCC were also significantly lower than those in patients with 1st RCC. Previous studies reported that 76.3% of patients with 2nd RCC had no obvious symptoms. In contrast, only 39.0% of patients with 1st RCC had no obvious symptoms. Moreover, the tumor size was smaller than 4 cm in 60.5% of patients with 2nd RCC, compared to 28.1% of patients with 1st RCC. These results are consistent with the findings in our study [9]. Patients with other non-renal malignancies tend to receive more frequent imaging examinations to increase surveillance of primary cancer, which contributes to the early detection of RCC. The fact that the proportion of partial nephrectomy in the patients with 2nd RCC was significantly higher than that in the patients with 1st RCC was also related to the smaller tumor size of the 2nd RCC.

Current guidelines recommend that renal surgery be performed in patients with localized RCC [11]. However, when choosing the treatment method for renal tumors in patients with 2nd RCC, the condition of the extrarenal malignancies needs to be considered, including tumor stage, life expectancy, and treatment complications. The European Association of Urology Guidelines on RCC indicates that elderly and comorbid patients with incidentally detected small renal masses can be managed by active surveillance (AS) [11]. However, considering that the sporadic RCC has a high risk of metastasis, AS of sporadic RCC should only be adopted in patients who cannot tolerate surgery [20]. A population-based study using the Surveillance, Epidemiology, and End Results (SEER)-9 database showed that local treatment modalities utilized with 2nd RCC were partial nephrectomy in 16% of patients, radical nephrectomy in 33% of patients, and ablation in 5.5% of patients, while 18% of patients received no local treatment [12]. In our results, the proportion of patients with 2nd RCC who received surgical treatment was 95.6%, which was higher than that reported in previous studies. This may be due to most patients with extrarenal malignancies in our study being stage I/II, which led to more patients with 2nd RCC choosing curable surgical treatment for the renal tumors.

Beisland et al. found that the OS of patients with 2nd RCC was worse than that of patients with 1st RCC [10]. Sato et al. showed that for patients with T1-2N0M0, the OS of patients with 2nd RCC was significantly worse than that of patients with 1st RCC [9]. These results are consistent with those of the present study. However, if extrarenal malignancies are excluded, Sato et al. showed that the CSS of patients with 2nd RCC was significantly longer than that of patients with 1st RCC [9]. In contrast, our study indicated that there was no significant difference in PFS and CSS between 2nd RCC and 1st RCC. A possible reason may be that 40.1% of patients with 1st RCC reported by Sato et al. were staged as T3/4, N1, or M1, while only 9.1% of patients with 1st RCC were staged as T3/4 or N1 in our study. These differences may have influenced the results of the two studies.

The prognostic factors of simple RCC include tumor stage, histopathological factors (such as nuclear grade, pathological type, sarcomatoid differentiation), and molecular markers [11]. In our study, univariate and multivariate analyses showed that for patients with 1st RCC, the influencing factors of OS included symptoms and stage, which was consistent with previous studies. However, the prognostic factors for 2nd RCC are more complex. Previous studies of multiple primary malignancies showed relationships between OS and the type of first primary malignancies or the interval between the two primary cancers [21]. In our study, univariate analysis showed that for 2nd RCC patients, OS was significantly correlated with the type of extrarenal malignancies and renal surgery, but there was no significant difference between OS and tumor interval, symptom, and stage. The prognosis of patients receiving renal surgery is better than that of patients without, and this may be related to the late-stage and poor prognosis of extrarenal malignancies, which leads to non-surgical treatment, resulting in a selection bias.

The present study has several limitations. Firstly, this study is retrospective. Hence, recall errors are possible and may affect the results. Secondly, this study excluded metastatic RCC patients both in the 2nd RCC group and the 1st RCC group; thus, there may be a certain selection bias. Thirdly, the follow-up time was relatively short, which may have affected the survival-related data analyses.

## Conclusion

In conclusion, there are some differences in the prognosis of 2nd and 1st RCCs which should be distinguished. Smoking is a high-risk factor for 2nd RCC. In the process of diagnosis, treatment, and follow-up of extrarenal malignancies, the occurrence of 2nd RCC should be monitored. The choice of treatment should be based on the conditions of the extrarenal malignancies and the RCC. Renal surgery should be performed when there are no obvious contraindications.

## Supplementary Information

**Additional file 1:** **Supplementary Table 1.** The origins of first primary malignancies in patients with second primary renal cell carcinoma (2nd RCC).

## Data Availability

The raw data supporting the conclusions of this article will be made available by the authors, without undue reservation.
