# Supplementary material for: A comprehensive analysis of renal cell carcinoma as first and second primary cancers
Source: World J Surg Oncol. 2022 Feb 27;20:57. doi: 10.1186/s12957-022-02493-6 (PMC8883617; doi:10.1186/s12957-022-02493-6)
Supplement: Supplementary file 1 — Additional file 1: Supplementary Table 1. The origins of first primary malignancies in patients with second primary renal cell carcinoma (2nd RCC). [file 12957_2022_2493_MOESM1_ESM.docx]

| Origins | | Second primary renal cell carcinoma |
| --- | --- | --- |
| Lung | | 23 (20.4%) |
| Colorectal | | 16 (14.2%) |
| Breast | | 12 (10.6%) |
| Gynecologic | | 11 (9.7%) |
| Thyroid | | 10 (8.8%) |
| Gastric | | 9 (8.0%) |
| Nasopharynx | | 9 (8.0%) |
| Esophagus | | 5 (4.4%) |
| Hematologic | | 4 (3.5%) |
| Urinary tract | | 3 (2.7%) |
| Tongue | | 2 (1.8%) |
| Soft tissue | | 2 (1.8%) |
| Prostate | | 1 (0.9%) |
| Liver | | 1 (0.9%) |
| Testis | | 1 (0.9%) |
| Larynx | | 1 (0.9%) |
| Parotid gland | | 1 (0.9%) |
| Brain | | 1 (0.9%) |
| Pancreas | | 1 (0.9%) |
| Tumor stage | I/II | 69 (61.1%) |
|  | III/IV | 21 (18.6%) |
|  | Missing | 23 (20.4%) |
| Receiving chemotherapy or  radiotherapy | Yes | 51 (45.1%) |
|  | No | 62 (54.9%) |

Supplementary table 1

The origins of first primary malignancies in patients with second primary renal cell carcinoma (2nd RCC)
